# Migraine, fibromyalgia, and depression among people with IBS: a prevalence study

**DOI:** 10.1186/1471-230X-6-26

**Published:** 2006-09-28

**Authors:** J Alexander Cole, Kenneth J Rothman, Howard J Cabral, Yuqing Zhang, Francis A Farraye

**Affiliations:** 1 Department of Epidemiology, Boston University School of Public Health, Boston MA, USA; 2 i3 Drug Safety, Auburndale, MA, USA; 3 Divsion of Preventive Medicine, Boston University School of Medicine, Boston MA, USA; 4 Department of Biostatistics, Boston University School of Public Health, Boston MA, USA; 5 Clinical Epidemiology Research and Training Unit, Boston University School of Medicine, Boston MA, USA; 6 Section of Gastroenterology, Boston University School of Medicine, Boston MA, USA

## Abstract

**Background:**

Case descriptions suggest IBS patients are more likely to have other disorders, including migraine, fibromyalgia, and depression. We sought to examine the prevalence of these conditions in cohorts of people with and without IBS.

**Methods:**

The source of data was a large U.S. health plan from January 1, 1996 though June 30, 2002. We identified all people with a medical claim associated with an ICD-9 code for IBS. A non-IBS cohort was a random sample of people with an ICD-9 code for routine medical care. In the cohorts, we identified all claims for migraine, depression, and fibromyalgia. We estimated the prevalence odds ratios (PORs) of each of the three conditions using the Mantel-Haenszel method. We conducted quantitative sensitivity analyses to quantify the impact of residual confounding and in differential outcome identification.

**Results:**

We identified 97,593 people in the IBS cohort, and a random sample of 27,402 people to compose the non-IBS comparison cohort. With adjustment, there was a 60% higher odds in the IBS cohort of having any one of the three disorders relative to the comparison cohort (POR 1.6, 95% CI 1.5 – 1.7). There was a 40% higher odds of depression in the IBS cohort (POR 1.4, 95% CI 1.3 – 1.4). The PORs for fibromyalgia and migraine were similar (POR for fibromyalgia 1.8, 95% CI 1.7 – 1.9; POR for migraine 1.6, 95% CI 1.4 – 1.7). Differential prevalence of an unmeasured confounder, or imperfect sensitivity or specificity of outcome detection would have impacted the observed results.

**Conclusion:**

People in the IBS cohort had a 40% to 80% higher prevalence odds of migraine, fibromyalgia, and depression.

## Background

Irritable bowel syndrome (IBS) is a functional disorder of the gastrointestinal tract, which results in the clinical symptoms of altered bowel habits and abdominal pain [1,2]. It is characterized by three specific forms: diarrhea-predominant, constipation-predominant, and alternating between diarrhea and constipation. While there is no diagnostic instrument to screen and diagnose people with the condition, the Rome II criteria are the established method of diagnosis for inclusion in clinical trials and by some clinicians [3].

People with IBS are reportedly more likely to have other disorders including migraine, fibromyalgia, and depression. These disorders are known in some literature as "affective spectrum disorders", or "functional somatic syndromes" [4,5]. This hypothesis, first proposed about fifteen years ago [4], was developed on the basis of observations that people treated with antidepressant therapy for these conditions responded favorably, since tricyclic antidepressants affect pain thresholds. Such findings promoted speculation that these disorders may actually be manifestations of the same underlying biologic mechanism. In an early study of this hypothesis that focused on people with fibromyalgia, 39% were found to have symptoms consistent with IBS, 45% had migraine, and 42% had chronic fatigue syndrome [6]. A more recent study showed a familial association among patients with major depressive disorder and fibromyalgia, IBS, and migraine [7]. Another study based on data from a Canadian clinic used latent class models to categorize reported symptoms from a survey, and suggested that a single diagnostic entity could replace the separate diagnostic criteria used to define fibromyalgia, IBS, depression and chronic fatigue syndrome [8]. Others have noted that the case definitions for each condition share similar diagnostic elements, and that perhaps IBS could be alternatively diagnosed as fibromyalgia or migraine, if a person had sought medical care from a rheumatologist or neurologist instead of a gastroenterologist [5]. Hypotheses surrounding fibromyalgia have been reinforced in data from a cross-sectional study in 1999 showing 32% of patients with IBS having symptoms of fibromyalgia, compared with 4% of patients without IBS [9]. Results from a more recent study showed fibromyalgia occurring in 20% of patients with IBS [10].

Data on the prevalence of depression in conjunction with IBS has been less clear. One study showed no association with psychological factors and health seeking behavior among IBS patients [11]. In contrast, a review of previously published work showed comorbidity of depression, anxiety, and somatoform disorders occurring in up to 94% of people with IBS [12].

Although the hypothesis surrounding the prevalence of these three disorders in association with IBS has been previously explored, many published studies have been based on data from clusters of case reports or from small clinical practices. Further limitations in these studies have been the lack of a reference cohort, and inability to fully control for potentially confounding variables. To address the validity concerns in previous research, we sought to examine the prevalence of other disorders, specifically migraine, fibromyalgia, and depression in cohorts of people with IBS and without IBS.

## Methods

### Data source

The context for this research was data from a large, national health insurance plan in the U.S. Coverage through the plan is available as an employment benefit and its members either are employed or are spouses and dependents of the employed subscriber. A research database contains automated insurance claims for medical services, including those provided by a physician or hospital, along with claims for prescriptions filled at an outpatient pharmacy. For this research, we used data from eight geographically diverse states with the largest concentration of health plan membership, primarily in the Midwest and south/southeastern United States.

### Study population

We identified all people with at least one medical claim associated with a diagnosis of irritable bowel syndrome between January 1, 1996 and June 30, 2002. The ICD-9 CM code corresponding to a diagnosis of irritable bowel syndrome is 564.1 [13]. To identify a group of people to serve as a comparison cohort, we identified a five percent simple random sample of all health plan enrollees that sought routine medical care with a physician and did not have an IBS diagnosis while enrolled with the health plan during the same period. The ICD-9-CM diagnosis code corresponding to a diagnosis of routine medical care is V70.0 [13]. We created datasets of all medical claims for people in the IBS and comparison cohorts, including those related to physician and hospital services, along with all outpatient pharmacy dispensing claims, and all dates of health plan enrollment.

### Identification of migraine, depression, and fibromyalgia

Among people in each of the two cohorts, we identified all medical and prescription claims relating to the occurrence of medical care for migraine, depression, and fibromyalgia. To decrease the probability of including claims for diagnostic testing, where the ICD-9 code may not reflect a definitive diagnosis (i.e. a "rule-out" diagnosis), we developed operational definitions for each of the three conditions. The operational definitions were as follows: Medical care for migraine was defined as claims for a physician visit or hospitalization associated with a diagnosis for migraine, and outpatient prescription claims associated with dispensing of an anti-migraine drug (ergot alkaloid or triptan). To be classified as having migraine, people were required to fulfill both the diagnosis and the prescription criteria. Medical care for depression was defined as claims for visits with a mental health provider associated with a diagnosis for depression. We defined medical care for fibromyalgia as claims for a physician visit or hospitalization associated with a diagnosis of fibromyalgia.

### Data analysis

To accommodate the nature of the dynamic populations in this study, and that migraine, fibromyalgia, and depression are chronic diseases without clear dates of onset, in these data we calculated the prevalence of each of the conditions in the following manner. For each month of the study period, we created a series of three dichotomous variables indicating whether the person received medical care for migraine, depression, or fibromyalgia, according to the operational definitions previously described. A person who fulfilled the operational definition for one of these conditions had the indicator variable for that variable flagged as one in that specific month. Once an individual fulfilled the operational definition in one of the months, all subsequent monthly indicator variables for that condition were flagged as one. The prevalence of each condition in the IBS and non-IBS cohorts was calculated as the fraction of people flagged with the condition as of the last month of the study period. Thus, for the purpose of estimating disease prevalence, a person with a short-lived depressive episode would be given the same weight as a person with depression lasting several years. To examine whether clustering any of the three conditions occurred in people, as has been suggested among people with IBS, we also calculated the prevalence of having two conditions and the prevalence of having all three conditions. For a measure of association, we used the prevalence odds ratio (POR). The crude prevalence odds ratio for each condition was calculated as the prevalence odds among the IBS cohort divided by the prevalence odds among the non-IBS cohort.

### Assessment of bias

Information bias stemming from the use of medical claims to identify each of the three conditions was a potential problem. People in the IBS cohort may be greater consumers of health care, and due to increased contact with health care providers, they could have a greater opportunity for a diagnosis of other diseases, including these three disorders. To address this source of possible bias, we created a variable representing the mean cost of all medical and pharmacy services reimbursed by the health plan during the study period per person-month of health plan enrollment, and treated it like a confounding variable in the analysis. Cost of medical and pharmacy services has been used in previous studies using these data as a proxy measure for disease severity [14,15]. Excluded from this cost measure was the cost of prescriptions and diagnoses associated with migraine, depression, and fibromyalgia. We stratified people according to quintiles of the distribution of mean monthly total medical cost. We also considered the effects of age and sex as possible confounding variables. We calculated the summary estimate of the prevalence odds ratio across strata of age and sex using the Mantel-Haenszel method.

### Sensitivity analysis

To quantify the possible effect of an unmeasured confounder on the observed prevalence odds ratios, we conducted quantitative sensitivity analyses based on simple methods [16]. This analysis estimated prevalence odds ratios for migraine following adjustment for an unmeasured dichotomous confounder that varied in prevalence and varied in the degree of association with migraine. We calculated adjusted odds ratios assuming that the prevalence of the confounder in the IBS cohort ranged from 10% to 90%, and that the odds ratio for the association between the unmeasured confounder and migraine varied from 2 to 10. We assumed that the prevalence of the unmeasured confounder in the non-IBS comparison cohort remained at 20%.

In another sensitivity analysis, we quantified the effect of outcome misclassification on the prevalence odds ratios. Although we developed operational definitions to identify the three disorders in medical claims data, there may be clinical inconsistency in the diagnosis of these conditions. These inconsistencies could include false-positive or false-negative diagnoses by a clinician. Using fibromyalgia as an example, we varied the hypothesized sensitivity and specificity of the outcome to assess the effect of possible diagnosis error. In these calculations, we assumed that the sensitivity and specificity of fibromyalgia detection was non-differential between the IBS and non-IBS cohorts. We calculated simulated prevalence odds ratios accounting for the hypothesized levels of misclassification.

### Privacy and confidentiality

All analyses and reporting for this research were performed using de-identified data with respect to Protected Health Information. Under the Health Insurance Portability and Accountability Act (HIPAA) of 1996, this research, using encrypted automated data, did not require approval by a Privacy Board or Institutional Review Board.

## Results

### Study population

We identified 97,593 people in the IBS cohort, and a random sample of 27,402 people to compose the non-IBS comparison cohort as a 5% random sample. The distribution of people in the two cohorts by age category and sex is shown in Table 1. Women constituted nearly 75% of the people in the IBS cohort, while about half of the people in the non-IBS cohort were women. The distribution of people in the two cohorts according to age category was nearly identical.

### Prevalence of migraine, fibromyalgia, and depression

The prevalence of migraine, fibromyalgia, and depression in the IBS and the non-IBS comparison cohorts are shown in Table 2. The table shows the prevalence of all three conditions, the prevalence of people with two-way combinations of conditions, and the prevalence of people with all three conditions. Among the IBS cohort, the prevalence of having at least one of the three disorders (migraine, depression, or fibromyalgia) was 264 per 1,000 people. In contrast, the prevalence of having at least one disorder was 118 per 1,000 people in the non-IBS cohort, corresponding to a pooled difference in the prevalence between the two cohorts of 46 per 1,000 people (95% CI 42 – 51). In both cohorts, depression was the most prevalent condition, occurring in 128 per 1,000 people in the IBS cohort, and 60 per 1,000 people in the comparison cohort (pooled prevalence difference 16 per 1,000, 95% CI 13 – 20).

### Adjusted prevalence odds ratios

Crude and pooled prevalence odds ratios for the three disorders are shown in Table 3. For all odds ratios, adjustment for age, sex, and the mean cost of medical services per person-month of health plan enrollment, substantially lowered the crude point estimate. Overall, people in the IBS cohort had a 60% higher odds of having one of the three conditions relative to the comparison cohort (pooled POR 1.6, 95% CI 1.5 – 1.7). For migraine, people in the IBS cohort had a 60% higher odds compared with people in the non-IBS cohort (pooled POR 1.6, 95% CI 1.4 – 1.7). The prevalence odds of fibromyalgia was 1.8-times greater in the IBS cohort relative to the comparison cohort (pooled POR 1.8, 95% CI 1.7 – 1.9). There was a 40% higher odds of depression in the IBS cohort relative to the comparison cohort (pooled POR 1.4, 95% CI 1.3 – 1.4).

### Sensitivity analysis

**Figure 1:**
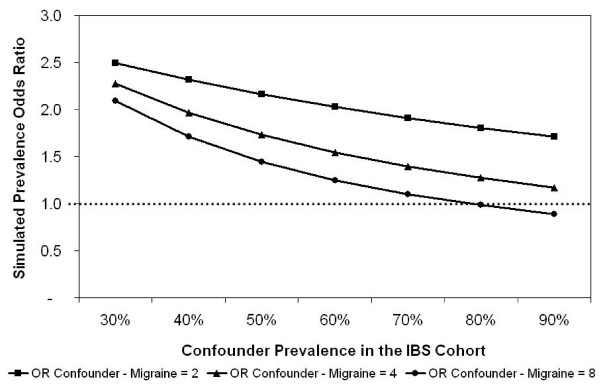
Sensitivity analysis of adjustment for an unmeasured dichotomous confounder on the prevalence odds ratio for migraine*. * Assumes a confounder prevalence of 20% in the non-IBS cohort.

Results of the sensitivity analysis of adjustment for an unmeasured dichotomous confounder are depicted graphically in Figure 1. The crude odds ratio for migraine was 2.8, and the analysis showed that a null association would have been observed after adjustment if there had been both a substantial difference in the prevalence of the confounder between the IBS and non-IBS cohorts and a strong association between the confounder and migraine. For example, an adjusted odds ratio of 1.0 for migraine would have been observed if the prevalence of the confounder was 80% in the IBS cohort, and the odds ratio between the confounder and migraine was 8.

Table 4 shows simulated crude prevalence odds ratios for fibromyalgia according to the hypothesized levels of non-differential sensitivity and specificity of outcome detection. The analysis indicated that imperfect sensitivity or specificity of fibromyalgia detection would have biased the observed prevalence odds ratio towards the null. The rows correspond to the hypothesized levels of sensitivity, and the columns correspond to the hypothesized levels of specificity. The observed crude prevalence odds ratio was 3.0, which is shown in the cell with 100% sensitivity and 100% specificity. The observed prevalence odds ratio for fibromyalgia would have been somewhat biased towards the null if the sensitivity of detection was less than 100%. For example, if the sensitivity of fibromyalgia detection was 75% while the specificity remained 100%, the simulated crude prevalence odds ratio would be 3.1. Bias towards the null at a more substantial level would have been introduced if the specificity of detection was less than 100%. If the specificity of fibromyalgia detection was 98% while the sensitivity remained at 100%, the simulated crude prevalence odds ratio would have been 4.4.

## Discussion

Within a large, national health insurance plan, we identified a cohort of 97,593 people with IBS, and a comparison cohort of 27,402 people that received routine medical services. Among people with IBS, the odds of having at least one of the three disorders was 60% greater compared with people without IBS. As suggested in previous research, we observed these three disorders to have a greater prevalence among people with IBS in comparison to those without IBS.

The crude, unadjusted, comparisons of the IBS to the non-IBS cohort provide an expectation of the prevalence of diagnoses of migraine, depression, and fibromyalgia in a group of people with IBS. This result can be useful for predicting use of health services, such as in an office setting. The adjusted results provide information relating to a causal effect of IBS, and addresses whether having IBS increase the prevalence of these conditions. The prevalence odds ratios were pooled across strata of age, sex, and quintile of the mean cost of medical services per person per month. The mean cost of medical services reflects the average consumption of health care services, and was intended to serve as a proxy measure for disease severity. Although disease severity itself is not a confounder, it creates a source of information bias that acts like a confounding variable owing to differential disease surveillance. Adjustment for these variables shifted the direction of the prevalence odds ratios towards the null for all of the point estimates. While the prevalence odds ratios were still elevated in the direction of excess prevalence in the IBS cohort, it is possible that using another variable that represented direct clinical measurements of disease severity could have improved adjustment for confounding. Such a shift from crude to pooled odds ratios suggests that improved adjustment in confounding could have resulted in a null association.

The sensitivity analyses illustrate some of the assumptions that underlie this study. Although residual confounding by an unmeasured variable can never be entirely ruled-out in epidemiologic research, the sensitivity analysis of adjustment for an unmeasured confounder indicated that the observed odds ratio would have been biased only if the prevalence of the confounding variable was extremely disproportionate between the IBS and non-IBS cohorts, and if there was a strong association between the confounder and migraine. This variable could be a common physiologic pathway of IBS and migraine. The sensitivity analysis for misclassification of fibromyalgia showed that variation in the correspondence of the insurance claims to actual clinical diagnoses could introduce bias. Sensitivity of fibromyalgia detection would have a small effect on the observed odds ratio. Variation in the specificity of fibromyalgia detection, on the other hand, would have greater effect on the observed odds ratio, resulting in more substantial bias towards the null than variation in the sensitivity. In interpreting the main findings of this study, we assume there are no unmeasured confounding variables and that the sensitivity and specificity of outcome detection is 100%. The sensitivity analyses, however, illustrate how violations of these assumptions could lead to a change in the interpretation of the results. The interpretation could change to one of no association between the IBS and non-IBS cohorts, if the presence and association with an unmeasured confounding variable was large enough. Alternatively, the interpretation could change to one of association of a considerable magnitude between IBS and the three disorders, if the degree of outcome misclassification was high.

The data used for this study were medical and pharmacy claims from a large, national health insurer. A limitation of the insurance claims data is that socioeconomic and quality of life measures, such as work absenteeism, which would be of considerable importance to an analysis of people with multiple comorbidities, are not obtainable. Further, since the people included in this study were those with commercial health insurance enrollment, the observed prevalence may be different than among people who may have different access to health services, such as those without health insurance, or other forms of insurance, such as government-sponsored Medicare or Medicaid plans.

All variables used in this study were derived from data elements ascertained from claims submitted for reimbursement, such as ICD-9 diagnosis codes and CPT-4 procedure codes. People in the IBS cohort were identified because they had a medical claim for health services associated with an ICD-9 diagnosis code of irritable bowel syndrome. The predictive value of an ICD-9 diagnosis code corresponding to IBS has been shown be around 80% [17,18], representing an IBS prevalence of 1.6% in this insured population [17]. However, there are limitations to using an insurance claims-based approach to classifying people with the disorder. One limitation is that were unable to ascertain clinical characteristics. This information might be obtainable through other primary data, such as through a physical examination or interview. These clinical characteristics might confound the relation we reported, or modify it. These data would also permit further evaluation of disorders and symptoms which are poorly reflected in insurance claims data, such as sleep disorders, chronic fatigue syndrome, and PTSD. Primary data would also permit confirmation of the diagnoses of IBS, fibromyalgia, depression, and migraine. For example, people with IBS are typically characterized according to their predominant symptom of constipation or diarrhea. In this analysis, however, we were unable to classify people according to their predominant symptom in the IBS cohort. We were therefore unable to assess whether there was heterogeneity in the prevalence of these conditions in the IBS cohort according the predominant symptom.

## Conclusion

People in the IBS cohort had a 40% to 80% higher prevalence odds of migraine, fibromyalgia, and depression in comparison to people without IBS.

## Competing interests

The author(s) declare that they have no competing interests.

## Authors' contributions

JAC conceived of the study, conducted the analysis, and drafted the manuscript. KJR participated in the design and helped to draft the manuscript. HJC participated in the analysis and helped to draft the manuscript. YZ participated in the analysis and helped to draft the manuscript. FAF participated in the design and helped to draft the manuscript. All authors read and approved the final manuscript.

## Pre-publication history

The pre-publication history for this paper can be accessed here:



## Figures and Tables

**Table 1:** Number of people in the IBS and non-IBS cohorts by age and sex, January 1996 through June 2002

	IBS cohort		Non-IBS cohort	
	No. of People	Percent	No. of People	Percent
Female				
18–29	13,791	19.6	3,291	23.2
30–39	16,435	23.3	3,409	24.1
40–49	19,263	27.3	3,811	26.9
50–64	18,837	26.7	3,430	24.2
65+	2,149	3.0	229	1.6
Total	70,475	100	14,170	100
				
Male				
18–29	5,224	19.3	2,434	18.4
30–39	6,972	25.7	3,094	23.4
40–49	6,993	25.8	3,759	28.4
50–64	6,925	25.5	3,638	27.5
65+	1,004	3.7	307	2.3
Total	27,118	100	13,232	100
				
Total				
18–29	19,015	19.5	5,725	20.9
30–39	23,407	24	6,503	23.7
40–49	26,256	26.9	7,570	27.6
50–64	25,762	26.4	7,068	25.8
65+	3,153	3.2	536	2.0
Total	97,593	100	27,402	100

**Table 2:** Prevalence of migraine, fibromyalgia, and depression, IBS and non-IBS cohorts, January 1996 through June 2002

	IBS cohort (n = 97,593)		Non-IBS cohort (n = 27,402)			
	Cases	Prevalence per 1,000	Cases	Prevalence per 1,000	Pooled Prevalence Difference*	(95% CI)
Migraine	5,890	60	611	22	5	(3 – 7)
Fibromyalgia	12,563	129	1,297	47	28	(25 – 31)
Depression	12,533	128	1,644	60	16	(13 – 20)
Any of the above	25,783	264	3,226	118	46	(42 – 51)
						
Depression and Fibromyalgia	2,149	22	146	5	2	(1.5 – 3)
Migraine and Fibromyalgia	1,032	11	66	2	1	(-0.2 – 2)
Depression and Migraine	924	10	74	3	0	(-0.2 – 0.3)
						
Depression, Migraine, and Fibromyalgia	549	6	20	1	0	(-0.5 – 1)

*Prevalence difference adjusted for categories of sex and quintile of mean medical costs per person-month of health plan enrollment.

**Table 3:** Prevalence odds ratios of migraine, fibromyalgia, and depression, January 1996 through June 2002

	Crude Prevalence		Pooled Prevalence	
	Odds Ratio	(95% CI)	Odds Ratio	(95% CI)
Migraine	2.8	(2.6 – 3.1)	1.6	(1.4 – 1.7)
Fibromyalgia	3.0	(2.8 – 3.2)	1.8	(1.7 – 1.9)
Depression	2.3	(2.2 – 2.4)	1.4	(1.3 – 1.4)
Any of the above	2.7	(2.6 – 2.8)	1.6	(1.5 – 1.7)
				
Depression and Fibromyalgia	4.2	(3.6 – 5.0)	2.0	(1.7 – 2.3)
Migraine and Fibromyalgia	4.4	(3.5 – 5.7)	2.0	(1.5 – 2.5)
Depression and Migraine	3.5	(2.8 – 4.5)	1.5	(1.2 – 1.7)
				
Depression, Migraine, and Fibromyalgia	7.7	(5.0 – 12.1)	2.6	(1.7 – 4.1)

*Mantel-Haenszel prevalence odds ratio pooled across categories of sex and quintile of mean medical costs per person-month of health plan enrollment.

**Table 4:** Simulated crude prevalence odds ratios for fibromyalgia according to hypothesized levels of observed non-differential sensitivity and specificity of outcome misclassification

	Specificity								
Sensitivity	100.0%	99.5%	99.0%	98.5%	98.0%	97.5%	97.0%	96.5%	96.0%
100.0%	3.0	3.2	3.5	3.8	4.3	5.1	6.2	8.3	13.2
95.0%	3.0	3.2	3.5	3.9	4.4	5.1	6.3	8.4	13.3
90.0%	3.0	3.2	3.5	3.9	4.4	5.1	6.3	8.4	13.4
85.0%	3.0	3.3	3.5	3.9	4.4	5.2	6.3	8.5	13.5
80.0%	3.0	3.3	3.6	3.9	4.5	5.2	6.4	8.5	13.6
75.0%	3.1	3.3	3.6	4.0	4.5	5.3	6.4	8.6	13.7
70.0%	3.1	3.3	3.6	4.0	4.5	5.3	6.5	8.7	13.8

## References

[B1] Horwitz BJ, Fisher RS (2001). The irritable bowel syndrome. N Engl J Med.

[B2] Drossman DA, Camilleri M, Mayer EA, Whitehead WE (2002). AGA technical review on irritable bowel syndrome. Gastroenterology.

[B3] Rome II Diagnostic Criteria for the Functional Gastrointestinal Disorders. http://www.romecriteria.org.

[B4] Hudson JI, Pope HGJ (1990). Affective spectrum disorder: does antidepressant response identify a family of disorders with a common pathophysiology?. Am J Psychiatry.

[B5] Wessely S, Nimnuan C, Sharpe M (1999). Functional somatic syndromes: one or many?. Lancet.

[B6] Hudson JI, Goldenberg DL, Pope HGJ, Keck PEJ, Schlesinger L (1992). Comorbidity of fibromyalgia with medical and psychiatric disorders. Am J Med.

[B7] Hudson JI, Mangweth B, Pope HGJ, De Col C, Hausmann A, Gutweniger S, Laird NM, Biebl W, Tsuang MT (2003). Family study of affective spectrum disorder. Arch Gen Psychiatry.

[B8] Robbins JM, Kirmayer LJ, Hemami S (1997). Latent variable models of functional somatic distress. J Nerv Ment Dis.

[B9] Sperber AD, Atzmon Y, Neumann L, Weisberg I, Shalit Y, Abu-Shakrah M, Fich A, Buskila D (1999). Fibromyalgia in the irritable bowel syndrome: studies of prevalence and clinical implications. Am J Gastroenterol.

[B10] Lubrano E, Iovino P, Tremolaterra F, Parsons WJ, Ciacci C, Mazzacca G (2001). Fibromyalgia in patients with irritable bowel syndrome. An association with the severity of the intestinal disorder. Int J Colorectal Dis.

[B11] Talley NJ, Boyce PM, Jones M (1997). Predictors of health care seeking for irritable bowel syndrome: a population based study. Gut.

[B12] Whitehead WE, Palsson O, Jones KR (2002). Systematic review of the comorbidity of irritable bowel syndrome with other disorders: what are the causes and implications?. Gastroenterology.

[B13] (2001). International Classification of Diseases, 9th Revision, Clinical Modification.

[B14] Loughlin JE, Cole JA, Dodd SL, Schein JR, Thornhill JC, Walker AM (2002). Comparison of resource utilization by patients treated with transdermal fentanyl and long-acting oral opioids for nonmalignant pain. Pain Med.

[B15] Cole JA, Loughlin JE, Ajene AN, Rosenberg DM, Cook SF, Walker AM (2002). The effect of zanimivir treatment on influenza complications: a retrospective cohort study.. Clin Ther.

[B16] Greenland S (1996). Basic methods for sensitivity analysis of biases. Int J Epidemiol.

[B17] Cole JA, Cook SF, Sands BE, Ajane AN, Miller DP, Walker AM (2004). Occurrence of colon ischemia in relation to irritable bowel syndrome.. Am J Gastroenterol.

[B18] Cole JA, Yeaw JM, Cutone JA, Kuo B, Huang Z, Earnest DL, Walker AM (2005). The incidence of abdominal and pelvic surgery among patients with irritable bowel syndrome. Dig Dis Sci.

